# A clinically concordant vulvar cancer patient-derived xenograft model retaining primary molecular signature for preclinical drug sensitivity assessment

**DOI:** 10.1515/med-2026-1419

**Published:** 2026-06-11

**Authors:** Yan Lin, Chunyan Chang, Na Liu, Dan Chai, Lihong Cai, Yuangiao He

**Affiliations:** Department of Obstetrics and Gynecology, Shangrao People’s Hospital, Shangrao, Jiangxi, China; Department of Basic Medical Sciences, School of Basic Medical Sciences, Nanchang University, Nanchang, China; Center of Laboratory Animal Science, Nanchang University, Nanchang, Jiangxi, China

**Keywords:** primary vulvar cancer, PDX, humanization identification, HDST, chemosensitivity

## Abstract

**Objectives:**

Establish and validate a patient-derived xenograft (PDX) model for vulvar cancer (VC). Evaluate the efficacy of commonly used clinical drugs both *in vitro* and *in vivo* using this model.

**Methods:**

Fresh primary vulvar cancer tumor tissues were then subcutaneously implanted into the scapular regions of immunodeficient mice, with tumor formation monitored and recorded in these mice. Hematoxylin and eosin (H&E) staining was performed to observe and compare the histopathological features of the primary and xenograft tumors, while immunohistochemical (IHC) staining was used to assess the consistency of protein expression profiles between the primary and xenograft tumors. Polymerase chain reaction (PCR) methods were employed to confirm the human origin of the xenograft tumors. Finally, the sensitivity of guideline-recommended chemotherapy drugs was evaluated using both HDST and *in vivo* efficacy studies, aiming to validate the drug responses observed *in vitro* with those seen *in vivo*.

**Results:**

Tumor tissue from a primary vulvar squamous cell carcinoma patient was successfully transplanted into the subcutaneous region of mice and stably passed to the fourth generation. H&E staining results showed that the PDX model retained the atypia and original histological features of the primary tumor cells. IHC staining further confirmed that the protein expression profile of the xenograft tumors in the PDX model was consistent with that of the primary tumor. PCR humanization identification confirmed that the tumor tissues were of human origin. HDST drug screening results, combined with *in vivo* efficacy experiments, validated the sensitivity of the PDX model to guideline-recommended chemotherapy drugs, demonstrating clinical consistency.

**Conclusions:**

We have successfully established a vulvar cancer PDX model. This model retains the fundamental molecular characteristics of the primary human vulvar cancer tumor, thus offering an advantageous approach for preclinical assessment of novel therapies and investigation into the disease’s pathogenesis.

## Introduction

Vulvar cancer is a rare gynecological malignancy originating from the skin or mucosa of the vulva, accounting for 2–5% of all female reproductive system cancers [1]. Squamous cell carcinoma (SCC) represents over 80 % of cases and is closely linked to human papillomavirus (HPV) infection, chronic inflammation, and genetic alterations [2], 3]. The disease predominantly affects elderly women, with more than half of diagnoses occurring in patients aged ≥60 years [4]. Current standard treatment relies on surgery combined with platinum-based chemotherapy or radiotherapy [5]; however, patients with advanced disease frequently experience limited efficacy – objective response rates range only from 20 % to 30 % – and severe toxicities such as myelosuppression and neurotoxicity [6], 7]. Critically, existing therapeutic strategies often overlook tumor heterogeneity and the complexity of the tumor microenvironment, highlighting an urgent need for more biologically faithful preclinical models to guide precision therapy.

Despite its clinical significance, vulvar cancer remains understudied due to its low incidence. To date, only one widely used cell line, SW962, has been reported – and it has been characterized solely through *in vitro* culture without any *in vivo* validation or animal modeling [8]. This lack of representative experimental systems severely hampers mechanistic investigation and drug development. Patient-derived xenograft (PDX) models, which involve direct engraftment of fresh tumor tissue into immunodeficient mice, have emerged as the “gold standard” in preclinical oncology because they preserve the histopathological architecture, molecular profile, and intratumoral heterogeneity of the original tumor [8], [9], [10]. While PDX models have been successfully established for other gynecologic cancers such as cervical and ovarian carcinomas [9], 10], their application in vulvar SCC remains virtually unexplored, with no systematically validated models reported to date.

Given these gaps – and acknowledging that our study is based on tumor tissue from a single patient, which inherently limits the generalizability of findings to the full heterogeneity of vulvar cancer – we conducted a proof-of-concept study. We hypothesized that a PDX model derived from this primary vulvar SCC case would recapitulate key histopathological and immunophenotypic features of the donor tumor, as assessed by H&E staining and immunohistochemistry (IHC), and reproduce the tumor’s known sensitivity to guideline-recommended chemotherapeutic agents (e.g., cisplatin, carboplatin, paclitaxel) *in vivo*. It should be emphasized that, due to limited tissue availability, comprehensive genomic analyses (e.g., targeted sequencing, copy number variation, or transcriptomic profiling) were not performed; thus, claims regarding molecular fidelity are confined to the markers evaluated in this study.

Therefore, this study addresses the following question: Can a patient-derived xenograft model be successfully established from a single case of vulvar squamous cell carcinoma and validated for histological, immunophenotypic, and functional fidelity with respect to established chemotherapy responses, thereby providing a foundational framework for future multi-case PDX studies that may explore novel therapeutics and resistance mechanisms.

## Materials and methods

### Patient clinical data

**Figure 1:**
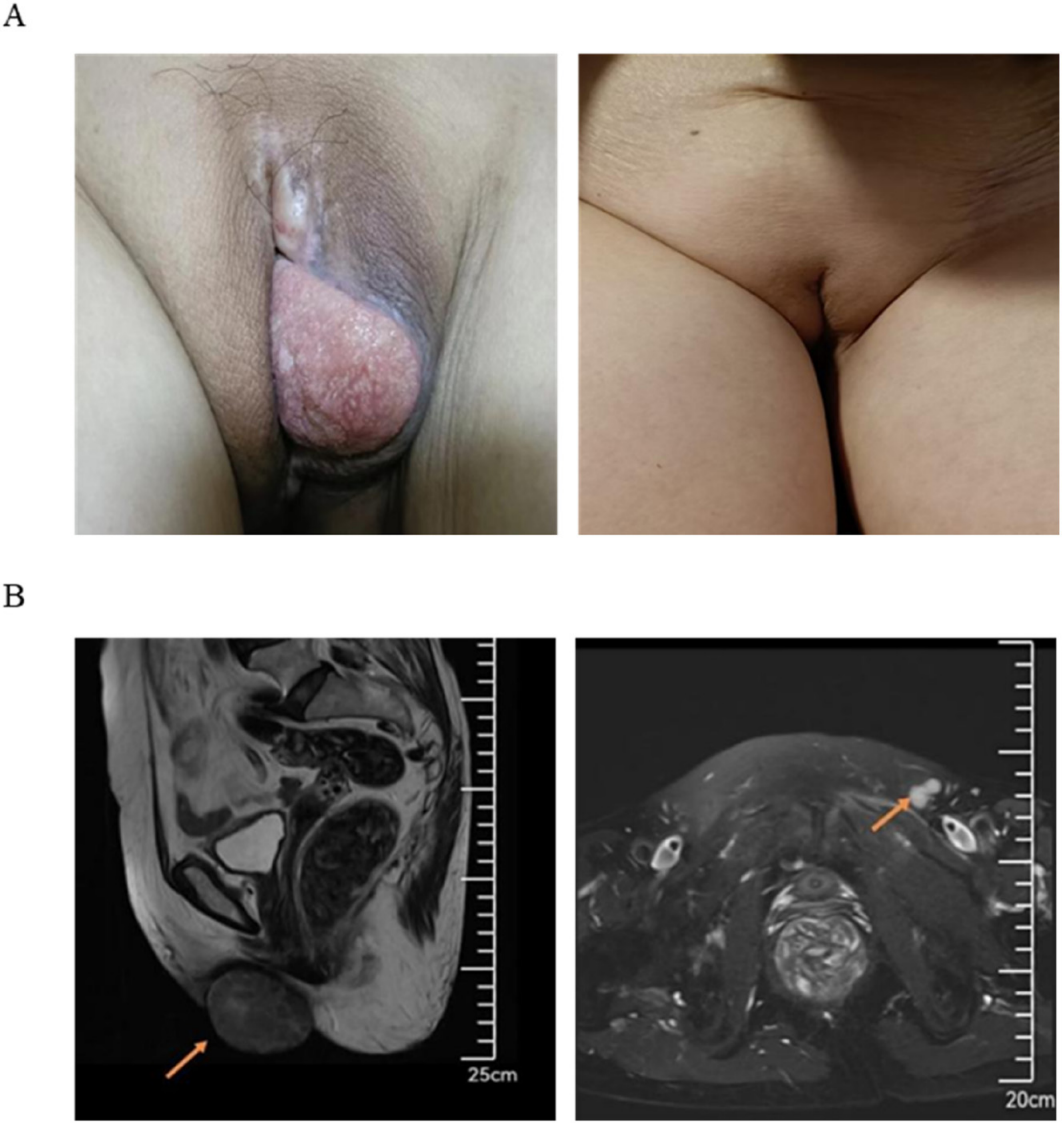
Clinical findings. (A) Preoperative and postoperative comparison of a vulvar cancer patient. (B) The provided MRI image shows the patient’s tumor tissue. Both the tumor mass and the enlarged lymph nodes are indicated with orange arrows.

A 40-year-old woman presented with a 2-year history of a pruritic, raised lesion of the vulva. Physical examination revealed a cauliflower-like, firm mass measuring 5 × 6 × 3 cm on the left labium majus, with well-defined borders and no tenderness. The lesion was accompanied by hypopigmented, gray-white skin changes extending to the anterior clitoral region. Postoperative comparison of the labia is provided in (Figure 1A). Magnetic resonance imaging (MRI) demonstrated an irregular mass predominantly involving the left labium majus, with sharply demarcated margins and ipsilateral inguinal lymphadenopathy, as shown in (Figure 1B). Pathological examination revealed a high-grade squamous intraepithelial lesion (VIN II) of the vulva. Immunohistochemical analysis showed negative expression of p16, positive staining for cytokeratin (CK), and approximately 55 % positivity for Ki-67.

Final histopathological diagnosis after surgery confirmed poorly differentiated vulvar squamous cell carcinoma with a stromal invasion depth of 11 mm and metastatic involvement of the left inguinal lymph node. The patient subsequently received adjuvant chemotherapy with paclitaxel and carboplatin and did not undergo radiotherapy.

### Laboratory animals

Female BALB/c nude mice and NOD/SCID mice, 6 weeks old, weighing 18–22 g, were purchased from Changzhou Cavens Model Animal Co., Ltd. Animal housing and all experimental procedures were conducted in the SPF animal facility at Nanchang Royo Biotech Co., Ltd.

### PDX model building

This study was approved by the Clinical Research Ethics Board of the First People’s Hospital of Shangrao City, and all patients provided written informed consent. All protocols adhered to the tenets of the Declaration of Helsinki. The samples were obtained from patients with pathologically confirmed vulvar cancer.

All human-related experimental procedures and methodologies were approved by the medical ethics committee of the First People’s Hospital of Shangrao City. Written informed consent was obtained from the individual(s) before sample collection. The approval date is 22 December 2022. The medical ethics committee number is Permit No. 2022078.

After obtaining informed consent from the patients, fresh tumor samples obtained from surgical resections were placed in tissue preservation solution and transported to the laboratory within 4 h at low temperature (2–8 °C). The tumor tissues were then subjected to viability staining and sterilization. Subsequently, the tissues were trimmed into pieces of 2 × 2 × 2 mm and implanted subcutaneously into the scapular region of nude mice using a trocar needle. This primary engraftment of patient-derived tumor tissue was designated as passage 0 (P0). Tumor formation was monitored weekly, and tumor volumes were measured twice per week once tumors were established. When P0 tumors reached a volume of 1,000–1,500 mm^3^, they were surgically excised, fragmented, and passage into a new cohort of nude mice to generate passage 1 (P1). Subsequent passages (P2–P5) were established by serial transplantation of tumor fragments from the previous passage into fresh recipient mice.

### H&E staining

The primary tumor specimens and P4 generation xenograft tissues were collected and fixed with 4 % paraformaldehyde for 24 h and then sent to the Department of Pathology of Shangrao People’s Hospital for paraffin embedding, and the tissue sections were stained with hematoxylin and eosin (H&E) to observe the histopathological morphology of the tumor.

### Immunohistochemical staining

The patient’s primary tumor paraffin block and P4 generation xenograft paraffin block were used for immunohistochemical staining, and all operations were completed by the Department of Pathology of Shangrao People’s Hospital. The expressions of CK, CK5/6, p53, p40, and Ki67 in the primary tumor paraffin block and P4 generation xenograft tissues were detected by immunohistochemistry using a Leica Bond-Max automated immunostainer.

### PCR humanized identification

To rule out the possibility that the subcutaneous xenograft tumor is derived from murine tumors, DNA was extracted from mouse xenograft tissues and PCR amplified for analysis [11].

### HDST *in vitro* susceptibility assay

Tumor tissue samples from the vulvar cancer (VC) PDX model were collected and subjected to viability screening. The more viable tissue fragments were selected and placed into a 96-well plate for *in vitro* culture. After 24 h of culture, the medium was supplemented with clinically recommended chemotherapeutic drugs according to the clinical chemotherapy guidelines. HDST *in vitro* tissue susceptibility testing. Detailed experimental methods can be found in our previously published work [12].

### Animal experiments

To validate the *in vitro* drug sensitivity results obtained from the HDST assay, selected single-agent drugs showing high tumor inhibition rates were further evaluated in NOD/SCID mice. *In vivo* efficacy was assessed by monitoring the general health status of the animals and measuring tumor inhibition rates, allowing for direct correlation with the HDST findings.

### Ethical approval

This study was approved by the Clinical Research Ethics Board of the First People’s Hospital of Shangrao City (Permit No. 2022078), and written informed consent was obtained from the patient prior to sample collection. All animal experiments were performed in accordance with institutional guidelines and approved by the Laboratory Animal Ethics and Welfare Committee of Nanchang Royo Biotech Co., Ltd (Approval No. RYE2023011901). All procedures involving animals complied with the ARRIVE guidelines, and every effort was made to minimize animal suffering.

### Statistical analysis

Data are presented as mean ± standard error of the mean (SEM). Comparisons between two groups were analyzed using an unpaired, two-tailed Student’s *t*-test, while comparisons among three or more groups were assessed by one-way analysis of variance (ANOVA) followed by Tukey’s post hoc test for multiple comparisons. All statistical analyses were performed using GraphPad Prism version 9.0 (GraphPad Software, San Diego, CA, USA). A p-value <0.05 was considered statistically significant. For *in vivo* drug efficacy studies, a minimum of three biologically independent mice per treatment group was used, in accordance with established standards for preclinical patient-derived xenograft (PDX) models.

## Results

### Successfully established the VC PDX model

**Figure 2:**
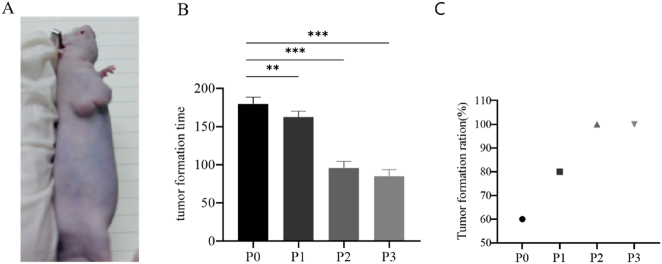
Establishment and characterization of a patient-derived xenograft (PDX) model of vulvar squamous cell carcinoma. (A) Representative image of a nude mouse bearing a subcutaneous PDX tumor following implantation of patient-derived tissue. (B) Tumor formation time across passages P0–P3. Data are shown as mean ± SD with n=5 mice per group. Statistical significance was determined by one-way ANOVA followed by Tukey’s post hoc test. p<0.01, p<0.001. (C) Tumorigenesis rate of subcutaneous tumor inoculation in P0∼P3 mice (n=5 mice per group).

In this study, tumor tissues directly obtained from patients were cut into approximately 10 small fragments and implanted subcutaneously into the bilateral scapular regions of five female mice. All animals remained in good condition two days after implantation. Two months post-implantation, round or oval, firm solid tumors emerged on the skin surface at the injection sites (Figure 2A). Around five months after tumor implantation, all mice developed subcutaneous tumors with a volume of approximately 1,000–1,500 mm^3^. The tumor tissues were then harvested and re-implanted into new recipient mice using the same procedure. The tumor formation time (Figure 2B) and tumor take rate (Figure 2C) across P0 to P3 generations were observed and recorded.

### Tissues from the PDX model and patients with cancer were consistent in morphological characteristics and protein expression

#### H&E and IHC

**Figure 3:**
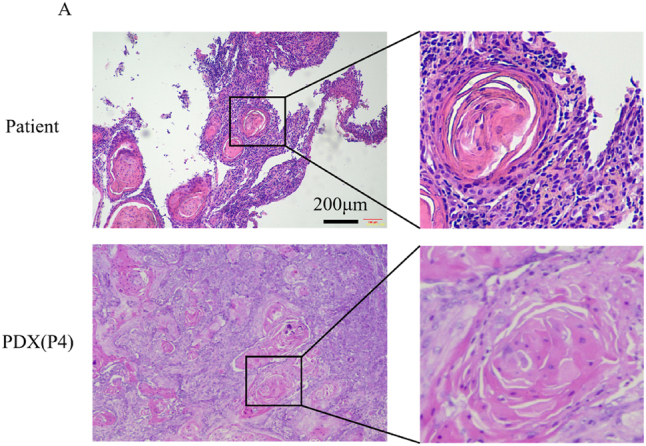
H&E staining of tumor pathological tissue sections of VC PDX model with matched clinical patients. Morphological characteristics were consistent in tumor tissue of patients and PDX model tissue.

**Figure 4:**
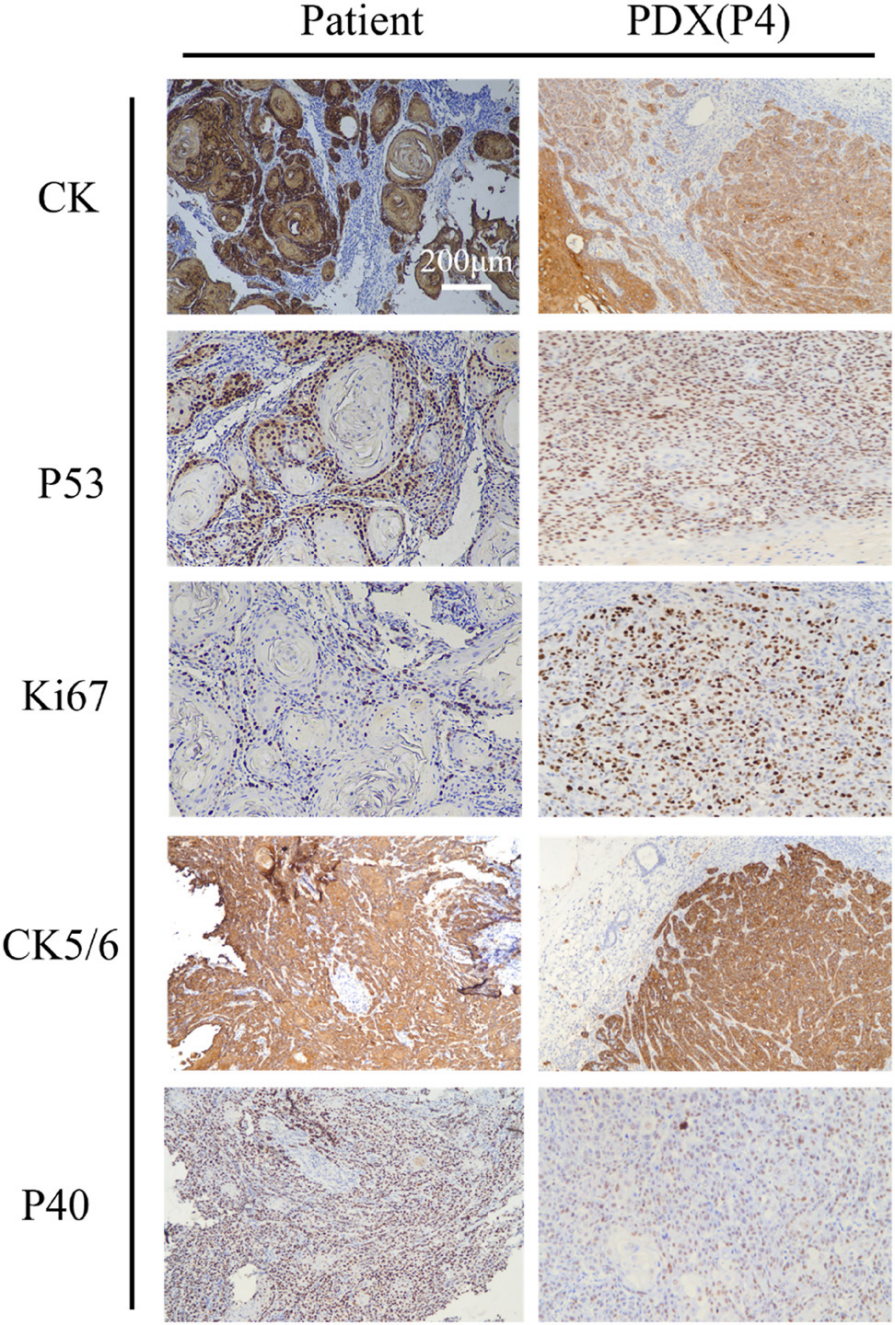
VC PDX model with matched clinical patient tumor tissue IHC. IHC results of the PDX(P4) tumor and the patient’s primary tumor tissue show: CK (+), CK5/6 (+), Ki67 (+, hotspot area: 60 %), p40 (+), p53 (+, mutation pattern).

The H&E stained tissue sections of the VC PDX (P4) model and the patient’s tumor tissue were placed under an optical microscope for observation: the tumor histopathological type, degree of differentiation, degree of keratinization, and nuclear atypia were compared. The VC PDX model has the same histopathological type of tumor as the clinical patients, with 80 % similarity in differentiation, keratinization and nuclear atypia. The PDX model has high histological loyalty with the corresponding clinical patients (Figure 3). To further confirm this conclusion, we also confirmed that the tumor tissues of the VC PDX model and the corresponding vulvar cancer markers of the corresponding patients: CK, CK5/6, P53, P40, and Ki67 were detected by immunohistochemistry, and the tumor tissues of the VC PDX model were highly consistent with the tumor tissues of clinical patients (Figure 4).

#### Organize humanized identification

**Figure 5:**
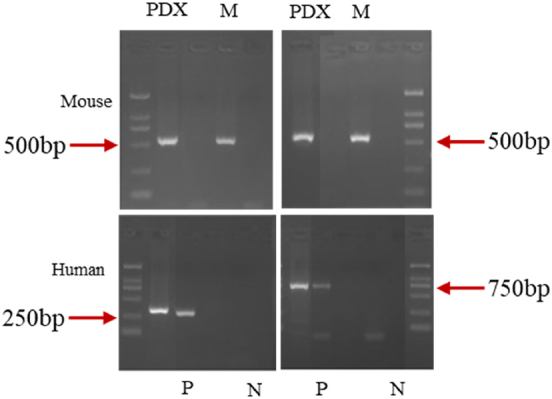
PDX model and patient cancer tissues were consistent in morphological characteristics and DNA expression. PDX(P4) tissue samples contained mouse and human target genes: PDX stands for PDX model tissue; P stands for patient tissue; M indicates a positive control of mouse origin, and N indicates a negative control.

DNA was extracted from fresh tumor tissues of the VC PDX model, and PCR was performed using human-specific DNA sequences and mouse-specific DNA sequences as primers to detect the species origin of the tumor tissue DNA in the PDX model. The results showed that the DNA of the PDX model tumor tissue contained both human and mouse DNA products (Figure 5).

### Clinical guideline-recommended drugs showed potent efficacy both *in vitro* and *in vivo*, with consistent tumor inhibition

#### *In vitro* efficacy

**Figure 6:**
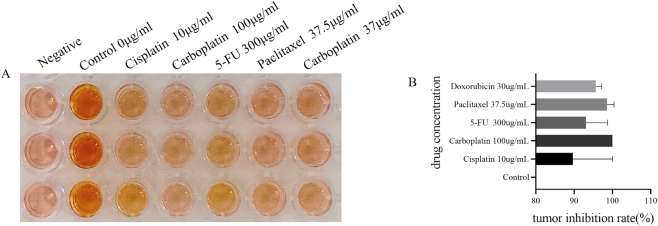
Clinical guideline-recommended drugs inhibited the viability of vulvar cancer cells *in vitro*. (A) HDST cell viability following treatment with carboplatin, cisplatin, 5-FU, paclitaxel, and doxorubicin was assessed using the CCK8 assay. (B) Statistical analysis revealed that carboplatin, cisplatin, 5-FU, paclitaxel, and doxorubicin significantly suppressed the activity of vulvar cancer cells, with tumor growth inhibition rates exceeding 80 % for all agents. All experiments were performed in triplicate, and data are presented as mean ± standard deviation (SD). Statistical differences between drug-treated groups and the control group were analyzed using one-way analysis of variance (ANOVA) followed by Tukey’s post-hoc test for multiple comparisons.

The tumor tissues of the VC PDX (P4) model were extracted for HDST susceptibility test, and the results showed that the inhibition rate of vulvar cancer tissues was quite different among different chemotherapy drugs (Figure 6).

#### *In vivo* efficacy

**Figure 7:**
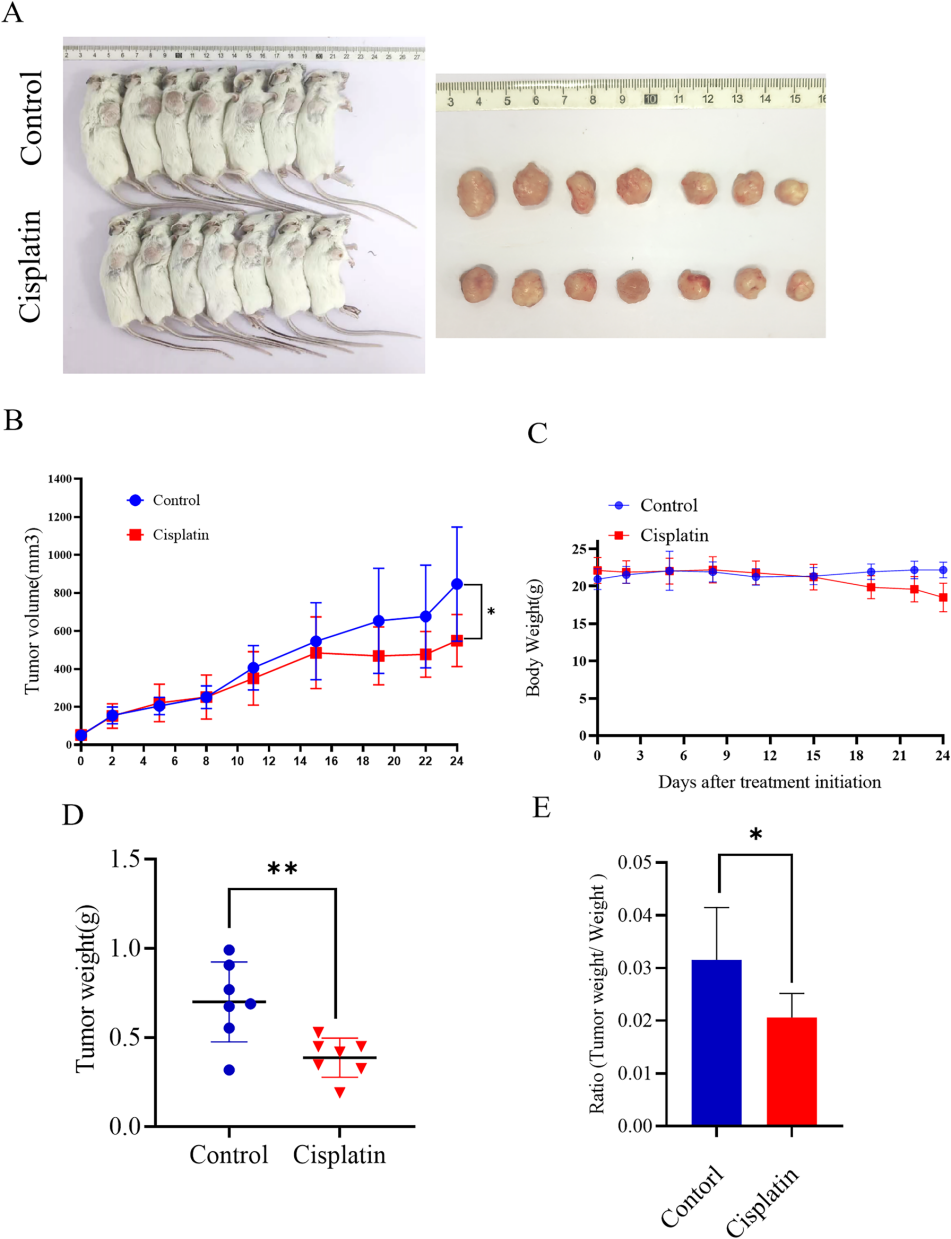
Cisplatin effectively inhibits tumor growth in a vulvar squamous cell carcinoma PDX model. (A) Representative images of nude mice and excised tumors from control and cisplatin-treated groups at the end of treatment. (B) Tumor volume over time during treatment. Data are shown as mean ± SD (n=7 per group). (C) Body weight changes in mice during treatment, indicating no significant toxicity. (D) Final tumor weights after treatment. (E) Ratio of tumor weight to body weight, reflecting relative tumor burden. Statistical significance was assessed using unpaired two-tailed student’s *t*-test: p<0.05, p<0.01.

Chemotherapeutic agents with high tumor inhibition rates identified through high-density spot test (HDST) *in vitro* drug sensitivity assays were selected for vivo efficacy experiments in mice. The validation of the HDST results was conducted using PDX (P5) tumors by assessing mouse general condition, tumor growth kinetics, and tumor inhibition rate (Figure 7).

## Discussion

In this study, we successfully established a patient-derived xenograft (PDX) model of vulvar squamous cell carcinoma (VSCC) from a single 40-year-old Han Chinese female with early-onset, HPV-independent disease, and validated its fidelity in histology, immunophenotype, and chemotherapy response. This work provides a foundational platform for preclinical research on this rare VSCC subtype.

The patient’s young age confers exceptional clinical relevance: VSCC typically affects women over 60, and early-onset cases (<40 years) are exceedingly rare, with limited biological or therapeutic data. Unlike elderly-onset VSCC – often driven by chronic inflammation, immune senescence, and HPV infection – early-onset tumors likely arise through distinct mechanisms such as germline mutations, epigenetic dysregulation, or intrinsic immune dysfunction. Consistent with this, our model is p16-negative, confirming an HPV-independent etiology and suggesting divergent oncogenic drivers. Moreover, younger patients may exhibit different tumor behavior – including proliferation, invasion, and drug metabolism – due to heightened systemic metabolic activity and intact tissue repair capacity, potentially leading to unique chemotherapy response profiles.

Histopathological and immunohistochemical analyses confirmed that the PDX model faithfully recapitulates key features of the primary tumor, including expression of squamous markers CK, CK5/6, and p40. Critically, the model preserves native tumor architecture and stromal components across serial passages – advantages absent in conventional systems. For instance, the widely used SW962 cell line, derived from a lymph node metastasis after long-term culture, suffers from genomic drift and lacks a tumor microenvironment [13], [14], [15], [16], [17]. Chemically induced models, while technically simple, fail to mirror human VSCC genetics or capture subtype-specific biology, especially for rare entities like early-onset, HPV-negative disease [18]. In contrast, our PDX model offers high biological loyalty, making it ideal for advanced functional studies.

Notably, this model aligns with emerging demands for spatially and molecularly authentic preclinical platforms. Technologies such as Perturb-DBiT enable spatially resolved CRISPR screening within intact tissue, revealing gene function heterogeneity in the native microenvironment – capabilities impossible in dissociated or cultured systems [19]. Similarly, multi-omic DBiT platforms (e.g., ARP-seq, CTRP-seq) can simultaneously profile chromatin states, transcriptomes, and proteomes at cellular resolution [20], 21]. Our PDX model, by retaining original tissue complexity, serves as a uniquely suited *in vivo* substrate for such cutting-edge investigations into the molecular underpinnings of early-onset VSCC [22], 23].

However, the most critical limitation is that the model derives from a single patient. All conclusions are restricted to this one case of early-onset, HPV-negative VSCC and cannot represent the broader heterogeneity of VSCC – which varies significantly by age, HPV status, stage, and molecular subtype [24], 25]. Consequently, findings on drug response or tumor biology lack generalizability and offer no universal guidance for clinical practice [26], 27]. Additionally, molecular validation was limited to IHC and species-specific PCR; comprehensive genomic or transcriptomic profiling was not performed due to tissue constraints. Thus, claims of “molecular fidelity” apply only to assessed protein markers, not genome-wide conservation [28]. Drug testing focused solely on standard chemotherapies (e.g., cisplatin, paclitaxel), without exploring novel agents, resistance mechanisms, or age-specific therapeutic vulnerabilities [29].

Therefore, the academic value of this study lies not in therapeutic innovation, but in proof-of-concept feasibility: we demonstrate, for the first time in China, that a PDX model of primary, early-onset VSCC can be established and maintained – a crucial step toward precision oncology for this understudied malignancy [30], 31].

To overcome the single-patient bottleneck, our central future direction is building a multicase PDX biobank encompassing diverse VSCC subtypes across age groups, HPV statuses, and clinical stages [29]. Such a cohort will enable comparative analyses to dissect how factors like age of onset shape tumor biology and drug sensitivity – addressing a major knowledge gap in early-onset VSCC [32], 33]. Integrated with high-throughput drug sensitivity testing (HDST), this platform will allow rapid screening of compound libraries followed by clinically relevant *in vivo* validation in matched PDX models [34]. Unlike our current focus on standard regimens, this pipeline could identify novel therapeutics and predictive biomarkers tailored to specific VSCC subtypes [35], 36].

Furthermore, coupling HDST with spatial functional genomics (e.g., Perturb-DBiT) will permit tissue-level mapping of drug-response determinants, while multi-omic DBiT profiling can uncover regulatory networks driving sensitivity or resistance in early-onset tumors [20], 21]. This integrated strategy bridges scalable screening and biologically faithful validation, accelerating translation for a disease urgently in need of better models and therapies [37], 38].

In conclusion, we report the first PDX model of primary, early-onset, HPV-independent VSCC in China, derived from a clinically distinctive case with a 2-year history of pruritus and exophytic lesions. While this model offers a valuable tool for probing the unique biology of this rare subset, its single-patient origin severely limits generalizability [26], 27]. We explicitly emphasize that findings apply only to this specific context and cannot inform broad VSCC management. Nevertheless, this proof-of-concept lays essential groundwork for a multicase PDX biobank – the indispensable next step toward molecular subtyping, biomarker discovery, and personalized therapy for VSCC [22], 39]. Given that spatial technologies increasingly rely on intact tumor ecosystems, our model represents a timely resource for advancing precision oncology in this neglected malignancy [23], 29].
